# A Rare Case of Primary Pulmonary Diffuse Large B-Cell Lymphoma Transformed from Marginal Zone Mucosa-Associated Lymphoid Tissue Lymphoma

**DOI:** 10.3390/medicina60060840

**Published:** 2024-05-21

**Authors:** Kajetan Kiełbowski, Dawid Kordykiewicz, Janusz Jesionka, Janusz Wójcik, Konrad Ptaszyński, Konstantinos Kostopanagiotou, Piotr Waloszczyk, Małgorzata Edyta Wojtyś

**Affiliations:** 1 Department of Thoracic Surgery and Transplantation, Pomeranian Medical University in Szczecin, Alfreda Sokołowskiego 11, 70-891 Szczecin, Poland; 2 Department of Pathology and Forensic Medicine, University of Warmia and Mazury Olsztyn, 11-082 Olsztyn, Poland; 3 Department of Thoracic Surgery, Attikon University Hospital of Athens, 12462 Athens, Greece; 4 Independent Laboratory of Pathology, Zdunomed, Energetyków 2, 70-656 Szczecin, Poland

**Keywords:** primary pulmonary lymphoma, MALT lymphoma, histologic transformation, DLBCL transformation

## Abstract

Primary pulmonary lymphoma is a rare neoplasm characterized by the proliferation of lymphoid tissue affecting the lungs. The most common subtype is marginal zone lymphoma of mucosa-associated lymphoid tissue (MALT). Rarely, a MALT lymphoma transforms into a diffuse large B-cell lymphoma (DLBCL). Treatment options include chemotherapy, radiotherapy, immunotherapy, and surgery. Here, we describe a patient with a primary pulmonary MALT lymphoma transforming into DLBCL. The purpose of this case report is to raise awareness of the relevant clinical and imaging features and to emphasize the need for a multidisciplinary approach to optimal management. In addition, we screened the PubMed and Embase databases for similar reports with a confirmed presence of transforming lymphoma within the lungs.

## 1. Introduction

Primary pulmonary lymphoma (PPL) represents approximately 0.5% of primary lung malignancies, less than 1% of lymphomas, and approximately 3% to 4% of extranodal lymphomas [1]. The most frequent type of PPL is marginal zone lymphoma of the mucosa-associated lymphoid tissue (MALT), accounting for up to 67% of PLL cases [2,3]. Gastric MALT lymphomas are associated with chronic inflammation due to long-term exposure to certain pathogens, such as *Helicobacter pylori* [4]. A similar infectious agent has not been identified in the pulmonary form [5], although some reports have suggested that an underlying interstitial lung disease could represent a potential risk factor [6]. In this article, we describe a patient with primary pulmonary MALT lymphoma that later transformed into diffuse large B-cell lymphoma (DLBCL). Interesting clinical and imaging features are presented in the following case, offering aid in the differential diagnosis of PPL versus non-small-cell lung cancer. Furthermore, we screened the PubMed and Embase databases for similar reports with a confirmed presence of transforming lymphoma within the lungs and compared their results with our case.

## 2. Case Presentation

**Figure 1:**
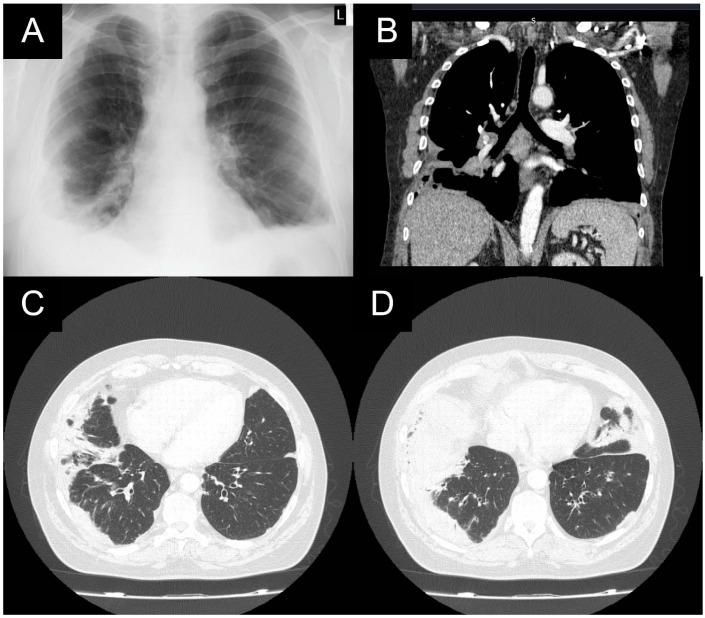
X-ray: coronal plane (**A**) from 2016; CT scan: coronal plane (**B**) and axial plane (**C**,**D**) from 2016. Extensive peripheral parenchymal infiltrates in both lower lobes, which are not typical for common pneumonia, in addition to mediastinal lymphadenopathy.

A 55-year-old male patient who had been experiencing an unproductive cough and low-grade fever for 4 weeks was admitted to the hospital after a diagnosis of culture-positive bilateral pneumonia. Apart from primary arterial hypertension and paroxysmal atrial fibrillation, the patient was a current non-smoker with 30 pack-years of smoking history and a positive family record of lung cancer. On admission, a computed tomography (CT) scan showed parenchymal consolidation and air bronchograms in the middle and both lower lobes, and marginal lymphadenopathy of the hilum, mediastinum, and axilla (Figure 1).

**Figure 2:**
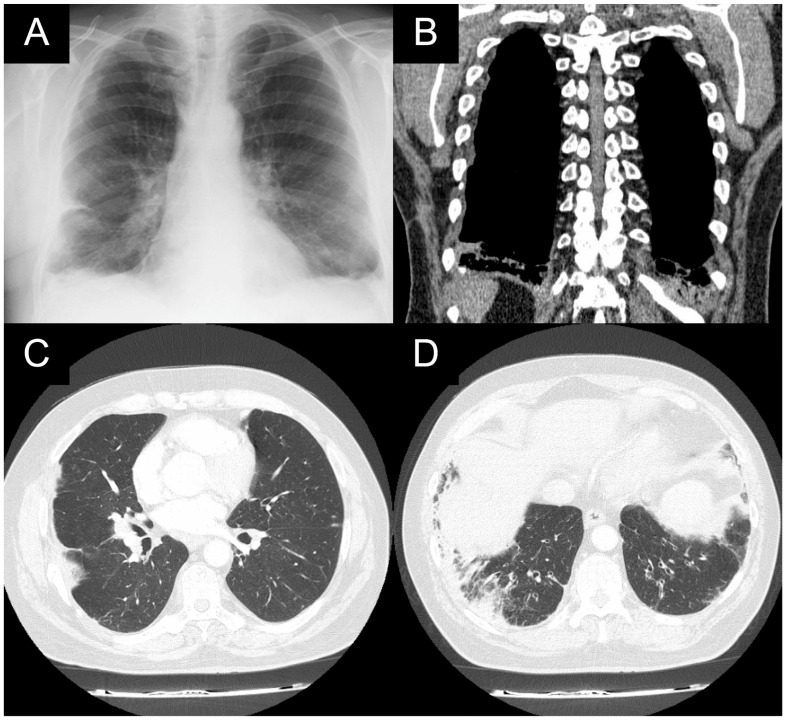
X-ray of the coronal plane (**A**) from 2017; CT scan: coronal plane (**B**) and axial plane (**C**,**D**) from 2017. Pulmonary infiltrates and consolidations in the radiological images.

The flexible bronchoscopy was unremarkable. His spirometry values for forced vital capacity (FVC) and forced expiratory volume in 1 s (FEV1) were 85% and 86.6%, respectively. The patient underwent a muscle-sparing mini-thoracotomy through the right eighth intercostal space and an atypical partial resection of the right lower pulmonary lobe. The histopathological diagnosis was lymphoid nodular hyperplasia (LNH). In 2017, pulmonary infiltrates were obvious on radiological imaging (Figure 2).

**Figure 3:**
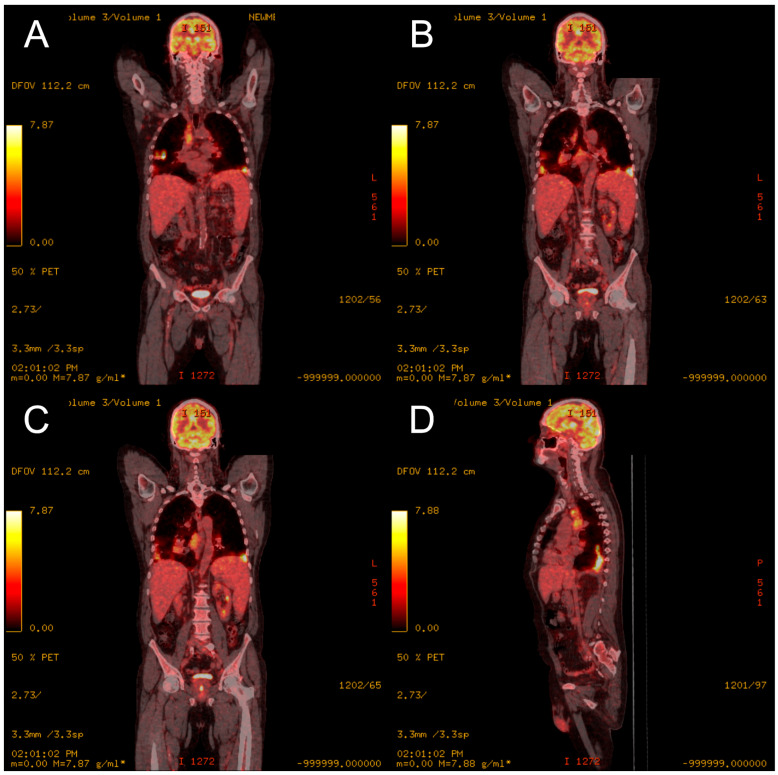
[¹⁸F]Fluorodeoxyglucose PET/CT from 2018 of the coronal plane (**A**–**C**) and sagittal plane (**D**), showing metabolically active mediastinal and hilar lymphadenopathy and pulmonary infiltrates.

An endobronchial ultrasound with transbronchial fine-needle aspiration was performed, but no neoplastic cells were found. Follow-up CT revealed the progression of pulmonary infiltrations. Positron emission tomography (PET) showed the elevated metabolic activity of the infiltrations (Figure 3). Furthermore, proliferative metabolic lesions were detected in the lymph nodes of the mediastinum, axillary cavities, and groin.

**Figure 4:**
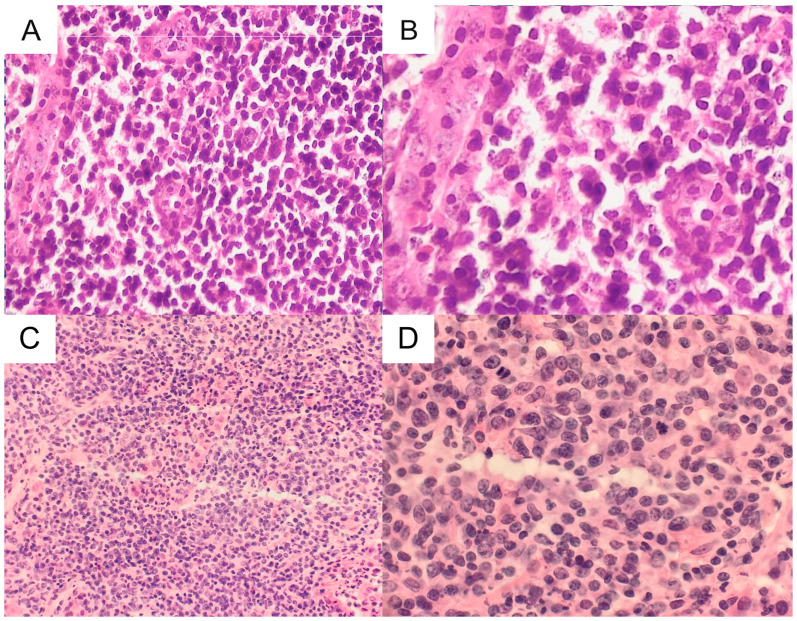
Histopathological specimens (representative hematoxylin-eosin–stained histological images)—(**A**) MALT lymphoma component. Small lymphoid cells, single plasma cells and monocytoid B-cells infiltrate small bronchiolar structures (original magnifications ×200). (**B**) MALT lymphoma component ((original magnifications ×400)). (**C**) DLBCL component ((original magnifications ×100)). (**D**) DLBCL component (original magnifications ×400).

**Figure 5:**
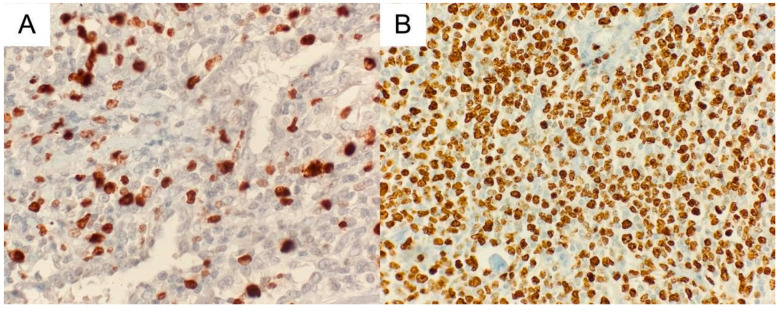
(**A**) MALT lymphoma component stained with Ki67 antibody (original magnifications ×400); (**B**) DLBCL component stained with Ki67 antibody original magnifications ×200).

**Figure 6:**
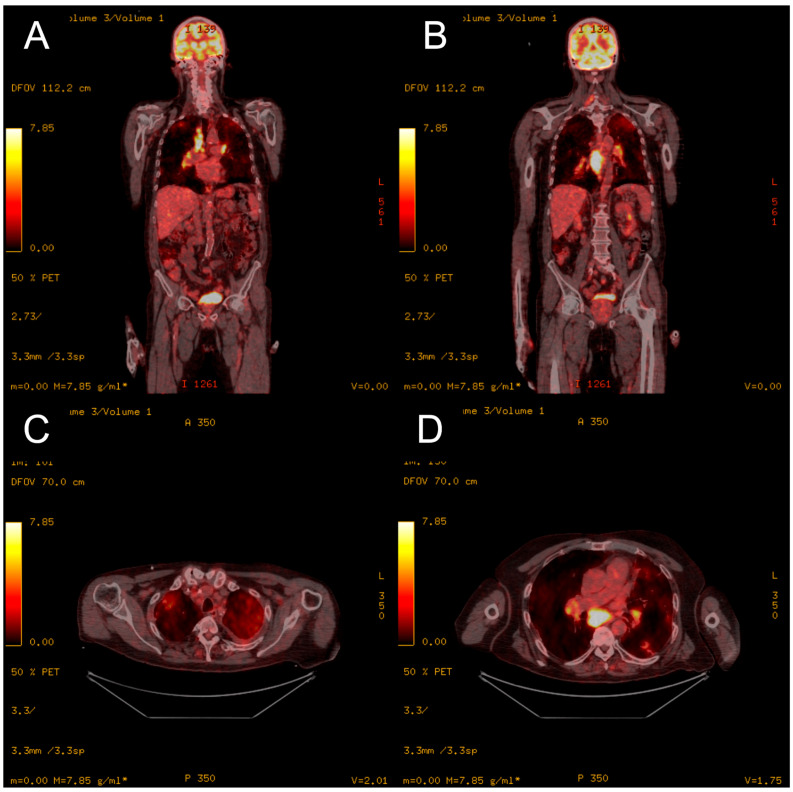
[¹⁸F]Fluorodeoxyglucose PET/CT from 2021 of the coronal plane (**A**,**B**) and axial plane (**C**,**D**), showing metabolically active mediastinal and hilar lymphadenopathy and pulmonary infiltrates.

Bronchofiberoscopy showed swollen mucous membranes and the presence of a purulent discharge throughout the bronchial tree. On re-evaluation spirometry, the FVC and FEV1 values were 68% and 64.6%, respectively. Video-mediastinoscopy revealed enlarged lymph nodes belonging to the sampled lymph nodes of the 4R group, which were collected for examination and were negative for malignancy. A neoplastic character was not confirmed. An approach through the left fifth intercostal space revealed a solid irregular infiltrate with a diameter of about 4 cm in the lingula, and irregular infiltrations in the 10th segment. The lingula was resected, and a histopathological examination showed multinodular infiltration of small and locally large lymphoid cells with the presence of lymphoepithelial structures. Local fields with the features of increased apoptosis of the cell groups were found. Extranodal marginal zone lymphoma of the MALT system with transformation into diffuse large B-cell lymphoma (DLBCL) was diagnosed (Figure 4). Neoplastic cells showed immunohistochemical expression of CD20 and CD79a and were negative for CD5, CD10, and CD23. The Ki-67 proliferation index was 60% (Figure 5). Postoperatively, the patient was referred to the hematology department, where chemotherapy was initiated. Three years later, the patient underwent autologous stem cell transplantation. The PET scan revealed metabolically active mediastinal and hilar lymphadenopathy with bilateral pulmonary infiltrations (Figure 6).

**Figure 7:**
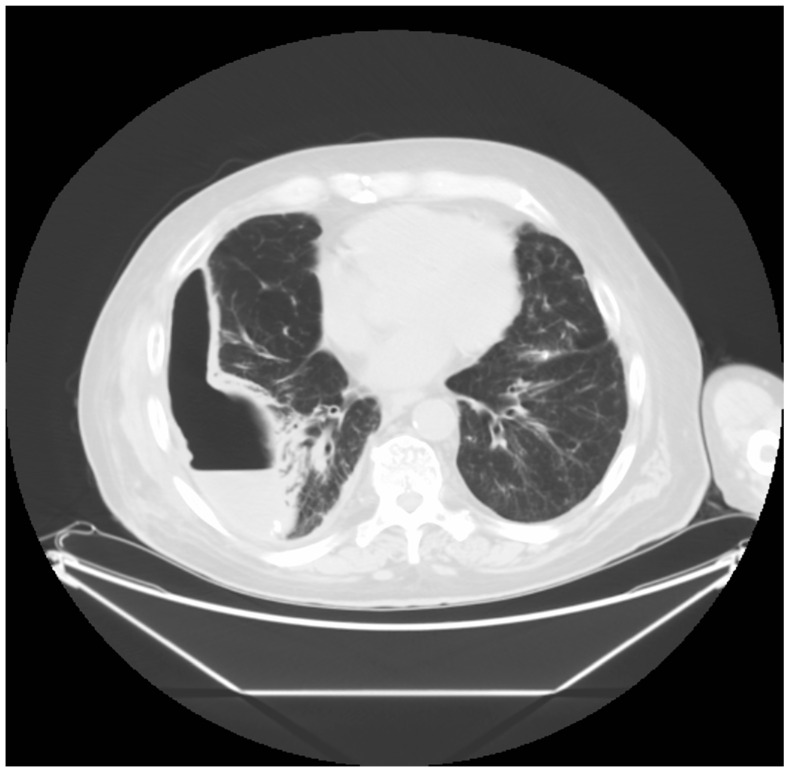
A CT scan image showing the trapped lung and free fluid, presumed to be a pyothorax or lung abscess. This condition was initially treated with guided percutaneous drainage.

Due to a suspicion of the recurrence of the lymphoma, a diagnostic thoracotomy was performed. A histopathological examination revealed multiple non-necrotizing granulomas and small lymphoid cells in the lymph nodes, while the pulmonary tissues exhibited post-inflammatory fibrosis and numerous granulomas. However, lymphoma was not detected. These findings, in conjunction with the clinical presentation, supported the diagnosis of sarcoidosis. One month later, the patient was readmitted to the hospital because of dyspnea, fever, cough, and oxygen saturation at 80% without oxygen supplementation. The inflammatory markers were elevated. Radiological examinations and cytobiochemical analysis of fluids confirmed pleural empyema and drainage was applied (Figure 7). Following the diagnosis of sarcoidosis, the patient was directed for additional diagnostic evaluation to the pulmonology department, which included a chest CT scan and an MRI evaluation of the heart. The results of the lung function tests showed a total lung capacity (TLC) of 69%, a residual volume (RV) of 50%, and a diffusing capacity of the lungs for carbon monoxide (DLCO) of 31%. The MRI evaluation of the heart revealed global hypokinesis of the left ventricular muscle, while the CT scan indicated a stable picture of changes. The patient is currently under continuous supervision at the lung disease clinic.

## 3. Discussion

Parenchymal pulmonary infiltrates can represent a hidden malignancy, and a histopathological examination is frequently essential to diagnose and stage potential cancer. Lymphoid infiltrates include LNH as well as subtypes of B-cell lymphomas, including MALT, small lymphocytic, or follicular lymphomas [7]. Additional histopathological examinations may be required if the lesions are located bilaterally or have a progressive character [8]. In the case presented here, LNH was the primary diagnosis, but subsequent examination revealed MALT lymphoma with transformation into DLBCL. This disease is defined by a clonal lymphoid proliferation that can involve the bronchi and parenchyma of one or both lungs with extrapulmonary involvement excluded at diagnosis [9]. The disease was restricted to the lungs, and the intrathoracic lymph nodes were not involved in the present case. Simultaneous involvement of pulmonary parenchyma and other organs, such as the stomach, also has been reported [10], but transformation of MALT to DLBCL within the lung is rare. We found only a few such cases when reviewing the literature available in the medical databases (Table 1) [11,12,13,14,15].

The disease is often associated with non-specific symptoms. According to Zhang et al. [2], 36% of patients are asymptomatic; 56% present with respiratory symptoms such as chest pain, cough, or dyspnea; and 27% report fever or weight loss. These symptoms also were reflected in our literature review, with cough [11,12,13,14] and dyspnea [11,12,13] as the most common patient-reported symptoms. Typical symptoms of lymphoma such as weight loss, night sweats, or persistent fever occurred in only two cases. Most patients had no medical history of lung disease, and only one patient had chronic obstructive pulmonary disease [14]. Because of the non-specific presentation, pulmonary lymphoma can be misdiagnosed as pneumonia [16]. Non-resolving pneumonia in a patient with a long history of smoking or lung cancer in the family, however, should always raise alertness about performing further diagnostics. In the case presented here, a CT showed bilateral consolidations with air bronchograms. Various abnormalities may appear on CT in patients with pulmonary MALT lymphoma. According to Deng et al. [17], the most common manifestations include consolidations (62%), followed by nodules (23%), masses (11%), and ground glass opacities (3.6%). In 36% of cases, thoracic lymphadenopathy is observed [17]. As compared with pneumonia, bronchiectasis and bulging of the interlobar fissure on CT imaging are more frequently observed in MALT lymphoma [18]. Imaging techniques such as CT and PET play important roles in both the diagnosis and monitoring of primary pulmonary lymphomas. They are crucial elements in diagnosis, staging, and planning treatment. PET/CT can detect areas of increased metabolic activity by providing precise anatomical information for targeting biopsies. Furthermore, they are essential for monitoring the response to treatment by evaluating changes in the size and characteristics of the lymphoma’s lesions over time and possible surgical complications such as pleural empyema.

The comprehensive approach involving multiple specialties is crucial. This includes utilizing radiological imaging, conducting histopathological examinations of tissue samples from surgical biopsies, and using immunohistochemical analysis. Integrating these diagnostic methods enables a thorough understanding of the tumor and facilitates the differentiation between lymphoma subtypes, which allows for appropriate treatment and prognosis to be determined. Lymphomas are best diagnosed with whole lymph node sampling or open biopsy of the extranodal lymphoma. Subsequent pathological evaluations may include histopathology, immunohistochemical study, flow cytometry, and molecular study. A needle biopsy approach frequently yields unreliable material, with usually insufficient or non-representative tissue sampling [19]. In our case, the core biopsy was non-diagnostic, and only intraoperative and then routine histopathological examination confirmed the MALT lymphoma. MALT lymphoma typically presents a monotonous proliferation of small to medium-sized lymphoid cells that infiltrate and expand the bronchial mucosa without destroying the underlying architecture. It often shows lymphoepithelial lesions, hallmark features where lymphocytes infiltrate the epithelial cells. These cells resemble those found in the marginal zone of lymphoid follicles; hence the name marginal zone lymphoma. A variable degree of plasma cell differentiation can be observed. Monocytoid B-cells with abundant pale cytoplasm are frequently seen in all marginal zone lymphomas, including MALT lymphoma. The typical immunohistochemical profile includes positive staining for B-cell markers such as CD20 and CD79a, confirming the tumor cells’ B-cell lineage. MALT lymphoma cells usually do not express CD5, CD10, and CD23, helping to distinguish them from other lymphomas that share these markers, such as mantle cell lymphoma and follicular lymphoma. Primary pulmonary DLBCL is characterized by the diffuse proliferation of large atypical B-cells. These malignant cells have large nuclei, often with prominent nucleoli, and a moderate to abundant cytoplasm. DLBCL exhibits positive immunostaining for B-cell markers such as CD20 and CD79a. However, DLBCL can further be characterized on the basis of the expression of additional markers such as CD10, BCL6, and MUM1/IRF4, which are used to categorize the lymphoma into either a germinal center B-cell-like (GCB) subtype or an activated B-cell-like (ABC) subtype. The expression of these markers in DLBCL not only aids in distinguishing it from MALT lymphoma but also has prognostic significance, as the GCB and ABC subtypes can have different treatment responses and outcomes. Borie et al. [20] suggested evaluating bronchoalveolar lavage in patients with suspected pulmonary MALT lymphoma, as the presence of a clonal B-cell population could help in the diagnosis. An immunohistochemical study is usually required to immunophenotype the cell population and identify their differentiation. Monoclonal B-cells are positive for CD79a, CD20, and Bcl-2 and negative for CD10, CD5, and cyclin D1. In addition, the Ki-67 proliferative index is usually low [21]. Maeshima et al. [22] reviewed data for 467 patients with MALT lymphoma in various locations and found histological transformation into DLBCL in 8%. In transformed lymphomas, 23% of patients had a Ki-67 index of >90%. In addition, through transformation, CD10 and BCL6 became positive in 9% and 59% of DLBCLs, respectively [22]. In another study by Kiesewetter and collaborators, the authors analyzed 379 patients with MALT lymphoma. In this cohort, 68% of patients developed extragastric disease. Twelve patients (3.2%) experienced a transformation towards an aggressive variant of lymphoma in a median time of 22 months. Interestingly, the authors observed that the majority of patients with transformed disease (67%) presented radiological signs of the lymph nodes’ involvement. An analysis of overall survival demonstrated that patients with transformed lymphoma had a significantly worse survival, as compared with non-transformed patients [23]. In reports that referred only to pulmonary MALT, the results were similar to the case presented here. The most frequently described immunohistochemical stains were CD20+, CD79+, Bcl-2+, CD5−, CD10−, and CD23− [11,12,13,14,15]. The Ki-67 index values in two patients with MALT also were low (10% and 20%), and, after transformation, depending on the patient and the study, ranged from 60% to 90% [14,15]. In recent years, case reports have begun to include information about the genetic basis of lymphoma’s transformation [14,15], describing mutations and rearrangements of several genes and their impact on potential treatment. The most studied genes include *IGHJ1*, *BCL6*, *NOTCH2*, *TNFAIP3*, *MYD88*, *TNFRSF*, *CREBBP*, *PIM1*, *MEF2B*, *BCL2*, and *MALT1-BIRC3*. These genetic alterations could potentially drive the evolution of MALT lymphoma towards a more aggressive DLBCL phenotype by promoting the cells’ proliferation, survival, and resistance to treatment. Furthermore, Huang and colleagues suggested that the overexpression of C-MYC may be associated with large tumor cells and predict an aggressive transformation [24]. Understanding the genetic basis of lymphoma’s transformation provides valuable insights into the underlying mechanisms.

Rituximab, a monoclonal antibody targeting CD20, is considered an effective first-line agent in monotherapy for pulmonary MALT lymphoma with significant antitumor activity [25]. This therapy was applied among the case studies analyzed here, except for two cases, in which a watch-and-wait strategy was used because of the lack of clear indications for treatment [14,15]. Targeted immunotherapy can also be used in combination with chemotherapy as a part of an R-CHOP regimen (rituximab + cyclophosphamide, doxorubicin, vincristine, and prednisone). In our article, we focused on showing the advantages of surgical treatment as part of multidisciplinary diagnostics. Xu et al. showed that in the early stage of the disease, the 5-year survival with pulmonary MALT lymphoma after surgical resection was 100% [26]. Targeted therapies seem to be the next element of treating lymphoma, but further research is necessary to determine their usefulness in the therapy of PPL. N-myristoyl transferase inhibitors represent the latest therapeutic agents that might be used in treating DLBCL [27].

## 4. Conclusions

The transformation of MALT to DLBCL is a rare situation that requires appropriate diagnostics, including awareness of this possibility and the use of immunohistochemistry, which may increase the recognition of cases. A surgical biopsy is diagnostic, especially when non-resolving pulmonary infiltrates raise the issue of differentiating between lung neoplasms and benign causes. During the course of treatment, PPL surgery may be required repeatedly in the diagnosis and treatment of therapeutic complications such as empyema.

## Figures and Tables

**Table 1:** Characteristics of included patients. NR—not reported.

First Author	Age	Sex	Symptoms	Transformation Time	MALT Immunohistochemistry	DLBCL Immunohistochemistry	MALT Ki67	DLBCL Ki67
Swarup [11]	59	Male	Dyspnea, cough; weight loss, night sweats, fevers	3 years	CD19(+), CD20(+), CD5(−), kappa(+)	NR	NR	NR
Diamantidis [12]	68	Male	Dyspnea, cough, hemoptysis, right chest pain	NR	NR	CD19(+), CD20(+), CD22(+), CD79a(+), CD5(−), CD10(−), CD23(−) (with focal transformation to high-grade)	NR	NR
Tiruneh [13]	63	Male		NR	NR	CD20(+), BCL2(+)	NR	NR
Zhou [14]	55	Female	Cough	3 years	LCA(+), CD20(+), CD79a(+), CD21(+), CD5(−), cyclinD1(−), CD43(−), CKpan(−), CK18(−)	CD3(−), CD5(−), CD43(−), CD20(+), CD79a(+), Pax-5(+), Bcl-2 50%(+), Bcl-6(+), CD10(+), MUM1(+), C-myc 40%(+), CD21(+), CD23(+), CD30(−), Cyclin D1(−), CD38(−), CD138(−), EBER(−)	20%	90%
Chen [15]	71	Male	Pain, leg edema, facial paralysis	2 years	CD3(−), CD20(+), Bcl-2(+), CD23(−), CD10(−), Bcl-6(−), CD5(−), Cyclin D1(−), Mum-1(−)	CD20(+), Bcl-2(+), CD23(−), CD10(−), Bcl-6(+), CD5(−), Cyclin D1(−), Mum-1(+), c-Myc (<10%+), SOX11(−)	10%	70%
Presented case	55	Male	Cough, weakness	2 years	NR	CD20(+), CD79(+), Bcl-2(+), CD5(−), CD10(−), CD23(−)	NR	60%

## Data Availability

The data presented in this study are available in this article.
